# Decreased coherence in the model of the dorsal visual pathway associated with Alzheimer’s disease

**DOI:** 10.1038/s41598-023-30535-w

**Published:** 2023-03-01

**Authors:** SiLu Yan, XiaoLi Yang, Hao Yang, ZhongKui Sun

**Affiliations:** 1 School of Mathematics and Statistics, Shaanxi Normal University, Xi’an, 710062 People’s Republic of China; 2 School of Mathematics and Statistics, Northwestern Polytechnical University, Xi’an, 710072 People’s Republic of China

**Keywords:** Applied mathematics, Diseases

## Abstract

Decreased coherence in electroencephalogram (EEG) has been reported in Alzheimer’s disease (AD) experimentally, which could be considered as a typical electrophysiological characteristic in AD. This work aimed to investigate the effect of AD on coherence in the dorsal visual pathway by the technique of neurocomputation. Firstly, according to the hierarchical organization of the cerebral cortex and the information flows of the dorsal visual pathway, a more physiologically plausible neural mass model including cortical areas v1, v2, and v5 was established in the dorsal visual pathway. The three interconnected cortical areas were connected by ascending and descending projections. Next, the pathological condition of loss of long synaptic projections in AD was simulated by reducing the parameters of long synaptic projections in the model. Then, the loss of long synaptic projections on coherence among different visual cortex areas was explored by means of power spectral analysis and coherence function. The results demonstrate that the coherence between these interconnected cortical areas showed an obvious decline with the gradual decrease of long synaptic projections, i.e. decrease in descending projections from area v2 to v1 and v5 to v2 and ascending projection from area v2 to v5. Hopefully, the results of this study could provide theoretical guidance for understanding the dynamical mechanism of AD.

## Introduction

Alzheimer’s disease (AD) is the most common neurodegenerative disease in elderly people. In the early clinical stage, memories and powers of concentration would be affected first. Follow-up clinical works found that AD would also impair language, executive and visual functions. Specifically, research on the visual cortex in AD showed that the spatial contrast sensitivity of the primary visual cortex in AD patients was significantly lower than that in elder adults with normal health^1^. Experimental results reported that the visual function of AD patients was selectively lost, including losses of color discrimination, stereoacuity, contrast sensitivity and backward masking, which were all caused by the lesion of primary and association visual cortices in AD patients^2^.

Visual-processing abnormality resulting from AD has attracted much attention in recent years. Notably, the dorsal visual pathway starts from area v1, passes through areas v2 and v3, and then projects to the middle temporal area (area v5) and inferior parietal lobule^3^. Often called ‘Where Pathway’, the dorsal visual pathway is involved in the processing of spatial position and related motion control like saccade and reaching. Several experimental studies have especially proposed that AD could affect the output pathway of the primary visual cortex: the dorsal visual pathway. For example, the area v5 of the dorsal visual pathway was detected by Positron Emission Computed Tomography (PET) and activated in healthy control (HC) with patterned flash stimulation at 1 Hz, but failed to be activated in AD patients^4^. Using functional magnetic resonance imaging (fMRI), patients with mild cognitive impairment (MCI) showed much less activation of the dorsal visual pathway in face and location matching tasks compared with those with HC^5^. Subsequent studies also showed abnormal functional connectivity in the dorsal visual area of AD and MCI by the technique of fMRI and mini mental state examinations^6,7^. What’s more, studies on the primary visual and visual association cortices of AD indicated that a loss of long-distance synaptic connections existed from areas v1 and v2 to area v5 in the dorsal visual pathway^8^.

The above works implied that AD could influence the dorsal visual pathway, particularly the long synaptic projections connecting the dorsal visual pathway, leading to severe restrictions on AD patients’ daily life. Thus, it is essential to search for some features which are beneficial for diagnosing AD before it causes greater damage to patients. Electroencephalogram (EEG) is considered a potential method of detecting AD in clinical practice. At present, lots of studies demonstrated that low coherence in EEG is a common phenomenon in AD and MCI patients. For instance, Locatelli et al. revealed that the coherence of the alpha band for AD patients in temporal, parietal and occipital lobes was significantly reduced compared with that for HC ones^9^. Hidasi et al. concluded that the most significant coherence decrease in AD was found in the alpha1 range^10^. Also, Wada et al. pointed out that the coherence of EEG signals between hemispheres in AD patients was reduced more significantly in the visual cortex^11^. Since the above works were mostly carried out in the lower rhythm band, Jelles et al. extended EEG coherence to the higher rhythm of the beta band and declared that the overall EEG coherence of AD patients was significantly lower in the beta frequency band than that of normal subjects^12^. The phenomenon that the coherence of EEG signals in AD patients was significantly lower than that in normal elderly was also reported in other works, and interested readers please refer to Refs.^13–16^. Thus, a decrease in EEG coherence has been regarded as one of the significant electrophysiological features of AD.

To better understand the change of EEG signals in experiments and reveal its intrinsic dynamic mechanism, much attention has been devoted to constructing some computational models on cortical areas, among which neural mass models (NMMs) are good candidates. In this model, the collective dynamical behaviour of neurons in the same population is described by a few state variables at the macroscopic level, given the assumption that the neurons in the same population receive same inputs and behave similarly. The concept of NMMs was first proposed by Wilson & Cowan^17^, Freeman^18^ and Lopes da Silva et al.^19^ in the 1970s. Therein, Wilson and Cowan constructed a simple neural mass model comprising excitatory and inhibitory neurons. Lopes da Silva et al. established a classical two-variable neural mass model which could produce alpha rhythm in the thalamus region. On this foundation, Freeman extended the two-variable model to the olfactory cortex and established a single cortical area model. Thereafter, Jansen et al. modified the two-variable model into a three-variable one containing three neural populations, i.e., pyramidal neurons and excitatory interneurons and inhibitory interneurons^20,21^. Importantly, according to the conclusion of physiological experiments in the hippocampal CA1 area, Wendling and Bartolomei proposed a four-variable model in the hippocampus by adding the fourth neuron population of fast inhibitory interneurons^22^. Zavaglia et al. further applied the model of Wendling to cortical regions on the basis of an experimental conclusion that fast inhibitory synapses also exist in a variety of cortical regions^23,24^. However, the above models could only produce a single rhythm in a single cortical area, which could not simulate real EEG signals composing various rhythms. In such case, Zavaglia et al. modeled the multi-cortical area by connecting three single cortical areas, in which various rhythms were emulated by changing the strengths and patterns of synaptic connections^25^. Recently, Mauro et al. introduced the self-inhibitory circuit of fast inhibitory interneurons to the above multi-cortical model, also simulated multiple rhythms and explored the transmission efficiency between cortical areas with different terminated neurons by the method of coherence function^26^. Although these studies have successfully constructed neural mass models to simulate EEG rhythms and power spectral densities, they did not take into account the pattern of laminar connections between cortical areas, which is considered to be a more comprehensive connection pattern in visual areas^27^. Thus, how the laminar connection pattern of the visual area could be introduced into the neural mass models to construct a model of the dorsal visual pathway would be discussed in this work.

As described above, previous experimental works have discussed the effect of AD on the dorsal visual pathway by combining medical diagnosis (mini mental state examinations, fMIR, PET and other imaging techniques) with statistical analyses^4–8^. However, different research methods and different subjects make it difficult to establish relationship between the results derived from various studies. The shortcoming is expected to be compensated by the technique of neurocomputation through constructing a biologically plausible neural mass model in the dorsal visual pathway. Motivated by these findings, this work aimed to establish a neural mass model with three interconnected cortical areas (v1, v2, and v5) according to the hierarchical organization of the cerebral cortex and the information flows of the dorsal visual pathway. The three cortical areas of v1, v2, and v5 are connected by long ascending and descending synaptic projections. Thereafter, the typical neuropathological change of loss of long synaptic projections in AD was simulated by reducing some synaptic connectivity parameters between different cortical areas. Further, the loss of long synaptic projections on coherence among different visual cortex areas was explored by means of power spectral analysis and coherence function.

This paper was divided into three parts: firstly, the neural mass model of the dorsal visual pathway and some preparatory presentation were introduced; secondly, the main results about how loss of long synaptic projections influenced coherence were discussed; finally, a brief conclusion and discussion of the whole work were presented.

## Model presentation and preliminary knowledge

**Figure 1:**
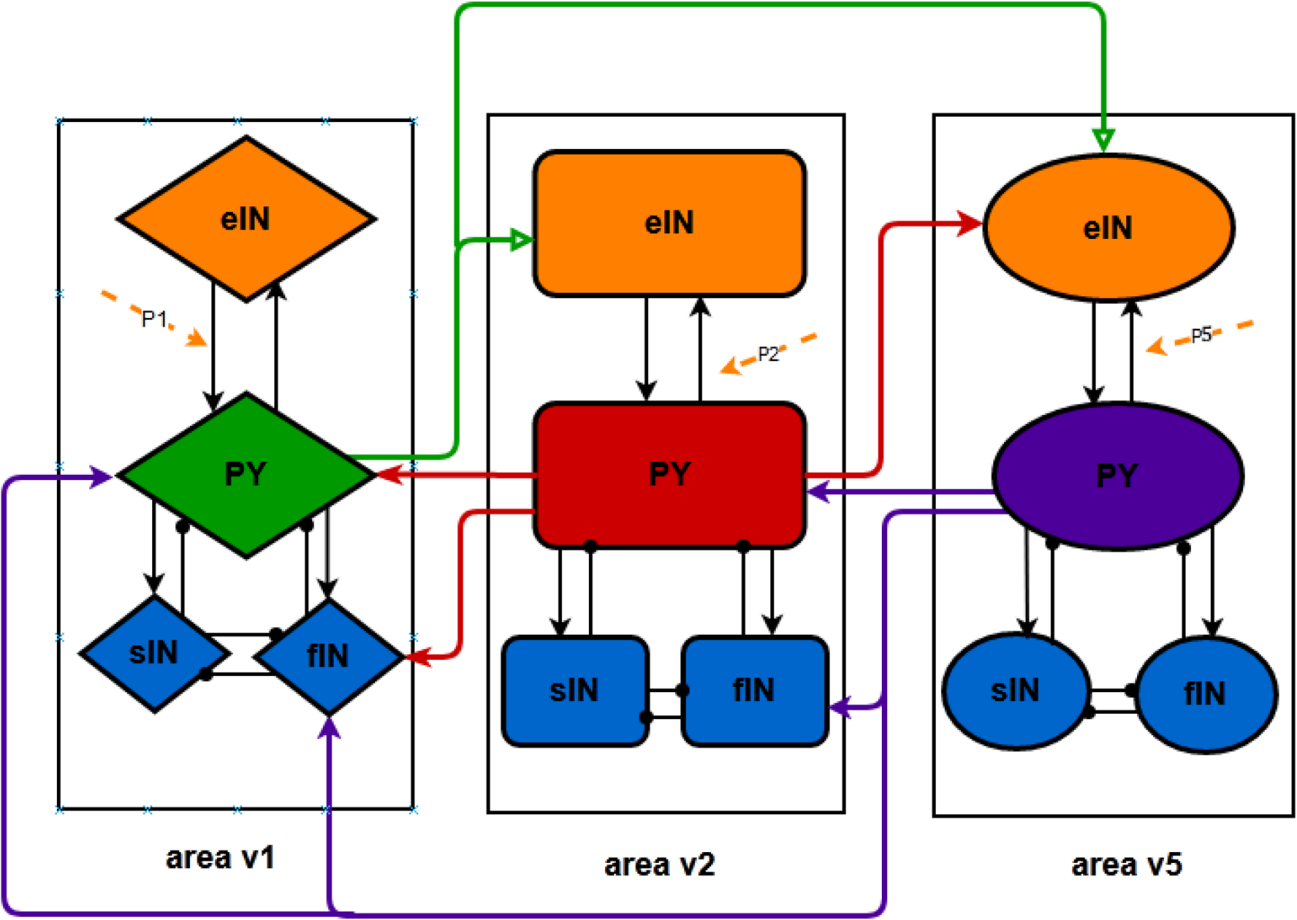
Diagram of the model sketch for the dorsal visual pathway. This neural mass model included three interconnected cortical areas of v1, v2, and v5. Each cortical area is composed of four neuron populations of PY, eIN, sIN and fIN. Black arrows represent excitatory synaptic connection and black rounds represent inhibitory synaptic connection in the single cortical area. Green arrows denote long synaptic projections from area v1 to v2 and v5; red arrows denote long synaptic projections from area v2 to v1 and v5; purple arrows denote long synaptic projections from area v5 to v1 and v2; orange-colored arrows denote Gaussian white noise which represents exogenous afferent from other connected areas not included in this model.

As mentioned in the Introduction, the dorsal visual pathway could be affected in AD. Thus, this section aims at constructing a more physiologically plausible neural mass model to describe the dorsal visual pathway. Since areas v2 and v3 have the same hierarchical organization and information flows^27^, some visual information like the direction of motion was transmitted directly from area v1 through area v2 to v5, while area v3 was omitted. For simplicity, this model includes only three cortical areas, i.e. areas v1, v2 and v5 connected by long synaptic projections. And each cortical area consists of four neuron populations of PY, eIN, sIN and fIN^23^. Based on the above assumptions and laminar patterns and connection principles in Felleman & Van-Essen^27^, the long synaptic projections (cortical-cortical connections) in the dorsal visual pathway mainly include ascending and descending projections. To be specific, the cerebral cortex is depicted by a standard six-layered structure, which contains supragranular and infragranular layers as well as granular layer 4^28^. The ascending projection originates from supragranular layers or both supragranular and infragranular layers and then converges on granular layer 4. The descending projection, their origination is located mainly in infragranular layers or both supragranular and infragranular layers, and finally terminates in all layers but layer 4. Further, based on the location of neurons in the neural mass model with the hierarchical pattern in David et al.^29^, PY neuron and inhibitory interneurons (sIN and fIN) could be predominately located in agranular layers (supragranular and infragranular layers) and layer 4 is occupied by eIN. For the dorsal visual pathway in this work, the long synaptic projections between the three cortical areas (areas v1, v2 and v5) could be constructed as follows. As illustrated in Fig. 1, for the forward pathway of the dorsal visual circuit, the PY neuron population of area v1 sends excitatory ascending projections to the eIN neuron population of areas v2 and v5 (the green arrow line), and that of area v2 also sends excitatory projection to area v5 (the rightward red arrow line). On the feedback pathway, the descending excitatory projection from the PY neuron population of area v5 is sent back to the fIN and PY neuron population of both areas v2 and v1 (the purple arrow line). Meanwhile, area v2 sends excitatory feedback to the fIN and PY neuron population of area v1 (the leftward red arrow line).

On the basis of the single cortical column proposed by Zavaglia et al.^23^, a single cortical area in this model is comprised of PY, eIN, sIN and fIN neuron populations. Therein, the PY neuron population sends excitatory connection to the other three neuron populations of eIN, sIN and fIN. Conversely, the neuron populations of sIN and fIN provide inhibitory feedback for the neuron population of PY. Meanwhile, the eIN neuron population sends an excitatory connection to the PY neuron population. Moreover, a mutually inhibitory effect between sIN and fIN neuron populations is introduced due to a basic disinhibitory circuit in the mammalian cerebral^30^. For details, the sketch of the neural mass model in the dorsal visual pathway is presented in Fig. 1. According to Fig. 1, the model equations governing each neuron populations can be written as follows.

**Area v1:**

$$\begin{gathered} \ddot{y}_{p1} = \dot{x}_{p1} = H_{e1} a_{e1} z_{p1} - 2a_{e1} x_{p1} - a_{e1}^{2} y_{p1} \hfill \\ z_{p1} = S\left( {v_{p1} } \right) \hfill \\ v_{p1} = c_{ep1} y_{e1} - c_{fp1} y_{f1} - c_{sp1} y_{s1} + y_{21} + y_{51} \hfill \\ \dot{y}_{21} = x_{21} \hfill \\ \dot{x}_{21} = H_{e1} a_{e1} \left( {k_{21}^{{}} z_{p2} (t - T)} \right) - 2a_{e1} x_{21} - a_{e1}^{2} y_{21} \hfill \\ \dot{y}_{51} = x_{51} \hfill \\ \dot{x}_{51} = H_{e1} a_{e1} \left( {k_{51}^{{}} z_{p5} (t - T)} \right) - 2a_{e1} x_{51} - a_{e1}^{2} y_{51} \hfill \\ \ddot{y}_{e1} = \dot{x}_{e1} = H_{e1} a_{e1} (z_{e1} + \frac{{p_{1} }}{{c_{ep1} }}) - 2a_{e1} x_{e1} - a_{e1}^{2} y_{e1} \hfill \\ z_{e1} = S\left( {c_{pe1} y_{p1} } \right) \hfill \\ \ddot{y}_{s1} = \dot{x}_{s1} = H_{s1} a_{s1} z_{s1} - 2a_{s1} x_{s1} - a_{s1}^{2} y_{s1} \hfill \\ z_{s1} = S\left( {c_{ps1} y_{p1} - c_{fs1} y_{f1} } \right) \hfill \\ \ddot{y}_{f1} = \dot{x}_{f1} = H_{f1} a_{f1} z_{f1} - 2a_{f1} x_{f1} - a_{f1}^{2} y_{f1} \hfill \\ z_{f1} = S\left( {c_{pf1} y_{p1} - c_{sf1} y_{s1} + y_{21} + y_{51} } \right) \hfill \\ \end{gathered}$$

**Area v2:**

$$\begin{gathered} \ddot{y}_{p2} = \dot{x}_{p2} = H_{e2} a_{e2} z_{p2} - 2a_{e2} x_{p2} - a_{e2}^{2} y_{p2} \hfill \\ z_{p2} = S\left( {v_{p2} } \right) \hfill \\ v_{p2} = c_{ep2} y_{e2} - c_{fp2} y_{f2} - c_{sp2} y_{s2} + y_{52} \hfill \\ \dot{y}_{52} = x_{52} \hfill \\ \dot{x}_{52} = H_{e2} a_{e2} \left( {k_{52}^{{}} z_{p5} (t - T)} \right) - 2a_{e2} x_{52} - a_{e2}^{2} y_{52} \hfill \\ \ddot{y}_{e2} = \dot{x}_{e2} = H_{e2} a_{e2} \left( {z_{e2} { + }\frac{{p_{2} }}{{c_{ep2} }}} \right) - 2a_{e2} x_{e2} - a_{e2}^{2} y_{e2} \hfill \\ z_{e2} = S\left( {c_{pe2} y_{p2} + y_{12} } \right) \hfill \\ \dot{y}_{12} = x_{12} \hfill \\ \dot{x}_{12} = H_{e2} a_{e2} \left( {k_{12}^{{}} z_{p1} (t - T)} \right) - 2a_{e2} x_{12} - a_{e2}^{2} y_{12} \hfill \\ \ddot{y}_{s2} = \dot{x}_{s2} = H_{s2} a_{s2} z_{s2} - 2a_{s2} x_{s2} - a_{s2}^{2} y_{s2} \hfill \\ z_{s2} = S\left( {c_{ps2} y_{p2} - c_{fs2} y_{f2} } \right) \hfill \\ \ddot{y}_{f2} = \dot{x}_{f2} = H_{f2} a_{f2} z_{f2} - 2a_{f2} x_{f2} - a_{f2}^{2} y_{f2} \hfill \\ z_{f2} = S\left( {c_{pf2} y_{p2} - c_{sf2} y_{s2} + y_{52} } \right) \hfill \\ \end{gathered}$$

**Area v5:**

$$\begin{gathered} \ddot{y}_{p5} = \dot{x}_{p5} = H_{e5} a_{e5} z_{p5} - 2a_{e5} x_{p5} - a_{e5}^{2} y_{p5} \hfill \\ z_{p5} = S\left( {v_{p5} } \right) \hfill \\ v_{p5} = c_{ep5} y_{e5} - c_{fp5} y_{f5} - c_{sp5} y_{s5} \hfill \\ \ddot{y}_{e5} = \dot{x}_{e5} = H_{e5} a_{e5} (z_{e5} + \frac{{p_{5} }}{{c_{ep5} }}) - 2a_{e5} x_{e5} - a_{e5}^{2} y_{e5} \hfill \\ z_{e5} = S\left( {c_{pe5} y_{p5} + y_{15} + y_{25} } \right) \hfill \\ \dot{y}_{15} = x_{15} \hfill \\ \dot{x}_{15} = H_{e5} a_{e5} \left( {k_{15}^{{}} z_{p1} (t - T)} \right) - 2a_{e5} x_{15} - a_{e5}^{2} y_{15} \hfill \\ \dot{y}_{25} = x_{25} \hfill \\ \dot{x}_{25} = H_{e5} a_{e5} \left( {k_{25}^{{}} z_{p2} (t - T)} \right) - 2a_{e5} x_{25} - a_{e5}^{2} y_{25} \hfill \\ \ddot{y}_{s5} = \dot{x}_{s5} = H_{s5} a_{s5} z_{s5} - 2a_{s5} x_{s5} - a_{s5}^{2} y_{s5} \hfill \\ z_{s5} = S\left( {c_{ps5} y_{p5} - c_{fs5} y_{f5} } \right) \hfill \\ \ddot{y}_{f5} = \dot{x}_{f5} = H_{f5} a_{f5} z_{f5} - 2a_{f5} x_{f5} - a_{f5}^{2} y_{f5} \hfill \\ z_{f5} = S\left( {c_{pf5} y_{p5} - c_{sf5} y_{s5} } \right) \hfill \\ \end{gathered}$$

In the foregoing equations, variables ${y}_{pi}$, ${y}_{ei}$, ${y}_{si}$ and ${y}_{fi}$ ($i = 1,2,5$) denote the postsynaptic membrane potential of PY, eIN, sIN and fIN neuron populations in areas v1, v2 and v5, separately.${c}_{mni}$ ($i = 1,2,5$) denotes the parameter of synaptic connection from presynaptic neuron population *m* to target neuron population *n* in areas v1, v2 and v5, where *m*, *n* are defined as follows,

$$m,n = \{ p,e,s,f\} ,$$

where $p,e,s,f$ represent PY, eIN, sIN and fIN neuron populations, respectively.${y}_{12}$ denotes the output of area v1 projecting to area v2 by the long synaptic projection ${k}_{12}$. Similarly, these definitions are also applicable to state variables:${y}_{15}$, ${y}_{25}$, ${y}_{21}$, ${y}_{51}$, ${y}_{52}$ and parameters of long synaptic projections:${k}_{15}$, ${k}_{25}$, ${k}_{21}$, ${k}_{51}$, ${k}_{52}$. ${P}_{i}$ ($i = 1,2,5$) denotes Gaussian white noise with mean value ${m}_{i}$ and variance ${\sigma }_{i}^{2}$. This noise represents exogenous afferent from other connected areas not included in this model. For different neuron populations, physiological parameters are also different. ${H}_{ei}$, ${H}_{si}$, ${H}_{fi}$ and ${a}_{ei}$, ${a}_{si}$, ${a}_{fi}$ ($i = 1,2,5$) are adopted to represent the average synaptic gain and the reciprocal of time constant for excitatory, slow and fast inhibitory synapses, respectively. Variables ${z}_{pi}$, ${z}_{ei}$, ${z}_{si}$, ${z}_{fi}$ ($i = 1,2,5$) represent the average discharge rates of PY, eIN, sIN and fIN neuron populations, respectively. Besides, the average firing rate ${z}_{mi}$ ($m = p,e,s,f$ and $i = 1,2,5$) is transformed into the average postsynaptic membrane potential ${v}_{mi}$ by a nonlinear sigmoid function $S( \bullet )$ as follows:

$$S\left( {v_{mi} } \right) = z_{mi} = \frac{{2e_{0} }}{{1 + {\text{e}}^{{ - rv_{mi} }} }} - e_{0}$$

where 2${e}_{0}$ represents the maximum firing rate of neuron populations^20^, and $r$ is the steepness parameter of the sigmoid function.

**Figure 2:**
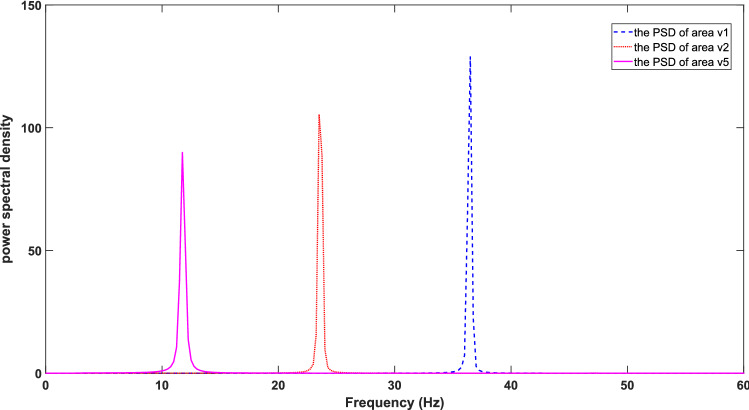
PSD of areas v1 (blue dotted line), v2 (red dashed line) and v5 (pink solid line) without long synaptic projections between the three cortical areas.

In this work, all parameters connecting different neuronal populations within cortical areas and the rest of the constant parameters are listed in Tables 1 and 2. To be specific, the synaptic connection parameters in Table 1 are based on Jansen et al.^21^, where all the connection parameters can be represented as a fraction of the connection parameter from PY to eIN. In Table 2, the value of parameters in areas v1 and v2 and the parameter of time delay *T* are chose based on Zavaglia et al.^25^. The value of parameters in area v5 and the parameter of Gaussian white noise are chose based on Zavaglia, et al.^23^. With these parameters in Table 2, the PSD of areas v1, v2 and v5 without long synaptic projections between three cortical areas is shown in Fig. 2. From this figure, it is clear that areas v1, v2 and v5 exhibited their intrinsic rhythms predominantly in gamma (30–48 Hz), beta (13–30 Hz)and alpha bands (8–12 Hz), respectively. For the area v1, some electrophysiological experimental works have confirmed that the area v1 could produce the gamma rhythm. For example, Maier et al. and Spaak et al. have investigated the association of the spontaneous gamma rhythm with layers in area v1 of macaques and found that the spontaneous gamma rhythm was present in all layers of the area v1^31,32^. For the area v5 (middle temporal area), Niedermeyer et al. have reported that in addition to the parieto-occipital alpha rhythm and the rolandic mu rhythm, there was a third alpha rhythm located in the midtemporal region^33^. For the area v2, the slow beta waves have been found to be located in the occipital lobe^34^. Thus, it can be seen that the positions of the peaks in the PSDs of the area v1, v2 and v5 in Fig. 2 are consistent with the results of the electrophysiological experimental works described above.

**Table 1:** Parameters of synaptic connections among different neuronal populations within each cortical areas.

	Origin	Target	Symbol	Value
Area v1	PY	eIN	$C_{pe1}$	65
		fIN	$C_{pf1}$	19.5
		sIN	$C_{{p{\text{s}}1}}$	19.5
	eIN	PY	$C_{ep1}$	52
	fIN	PY	$C_{fp1}$	52
		sIN	$C_{fs1}$	6.5
	sIN	PY	$C_{sp1}$	19.5
		fIN	$C_{sf1}$	6.5
Area v2	PY	eIN	$C_{pe2}$	80
		fIN	$C_{pf2}$	24
		sIN	$C_{pe2}$	24
	eIN	PY	$C_{ep2}$	64
	fIN	PY	$C_{fp2}$	64
		sIN	$C_{fs2}$	8
	sIN	PY	$C_{sp2}$	24
		fIN	$C_{sf2}$	8
Area v5	PY	eIN	$C_{pe5}$	59
		fIN	$C_{pf5}$	17.7
		sIN	$C_{ps5}$	17.7
	eIN	PY	$C_{ep5}$	47.2
	fIN	PY	$C_{fp5}$	47.2
		sIN	$C_{fs5}$	5.9
	sIN	PY	$C_{sp5}$	17.7
		fIN	$C_{sf5}$	5.9

**Table 2:** Other basal parameters in the model of dorsal visual pathway.

Symbol	Unit	Module	Value	References
$s_{0}$	mV	v1/v2/v5	6	Zavaglia et al.^25^
$e_{0}$	s^−1^	v1/v2/v5	2.5	Zavaglia et al.^25^
$r$	mV^−1^	v1/v2/v5	0.56	Zavaglia et al.^25^
$H_{e1}$	mV	v1	5.6	Zavaglia et al.^25^
$H_{s1}$	mV	v1	3.8	Zavaglia et al.^25^
$H_{f1}$	mV	v1	173.1	Zavaglia et al.^25^
$a_{e1}$	ms^−1^	v1	110	Zavaglia et al.^25^
$a_{s1}$	ms^−1^	v1	40	Zavaglia et al.^25^
$a_{f1}$	ms^−1^	v1	790	Zavaglia et al.^25^
$m_{1}$		v1	100	Zavaglia et al.^23^
$\sigma_{1}^{2}$		v1	60	Zavaglia et al.^23^
$H_{e2}$	mV	v2	5.2	Zavaglia et al.^25^
$H_{s2}$	mV	v2	4.5	Zavaglia et al.^25^
$H_{f2}$	mV	v2	57.1	Zavaglia et al.^25^
$a_{e2}$	ms^−1^	v2	85	Zavaglia et al.^25^
$a_{s2}$	ms^−1^	v2	30	Zavaglia et al.^25^
$a_{f2}$	ms^−1^	v2	350	Zavaglia et al.^25^
$m_{2}$		v2	100	Zavaglia et al.^23^
$\sigma_{2}^{2}$		v2	60	Zavaglia et al.^23^
$H_{e5}$	mV	v5	2.7	Zavaglia et al.^23^
$H_{s5}$	mV	v5	3.2	Zavaglia et al.^23^
$H_{f5}$	mV	v5	39	Zavaglia et al.^23^
$a_{e5}$	ms^−1^	v5	40	Zavaglia et al.^23^
$a_{s5}$	ms^−1^	v5	20	Zavaglia et al.^23^
$a_{f5}$	ms^−1^	v5	300	Zavaglia et al.^23^
$m_{5}$		v5	100	Zavaglia et al.^23^
$\sigma_{5}^{2}$		v5	60	Zavaglia et al.^23^
$T$	ms	v1/v2/v5	10	Zavaglia et al.^25^

The numerical emulation method for the above differential equations is Euler solver in Matlab R2021a simulating environment. All equations were calculated for 600 s, and the time step of these equations is 0.002 s. Therein, the simulation results of the first 30 s were abandoned for acquiring stable state data. In previous work, EEG signals could be considered as the average depolarization of pyramidal neurons^29^. Thus, in this work it was assumed that the output of cortical areas originated from pyramidal neurons, which were presented by the variables of the average postsynaptic membrane ${v}_{pi}$ $(i = 1,2,5)$. In addition, the setting of model parameters followed the value in Tables 1 and 2. As for the estimation of PSD, firstly, the output of equations needed to be filtered by the Butterworth filter of order 5 with a lower cutoff frequency of 3 Hz and a higher cutoff frequency of 60 Hz. And then, Welch’s averaged modified periodogram method with a 50% overlapping hamming window was chosen to calculate PSD. Finally, the average value of PSD was computed 50 times to ensure statistical accuracy.

This work also needed to estimate the coherence function of two outputs in this model. The coherence function between two signals ($x \left(t\right)$ and $y (t)$) is defined as follows^25,31^:

$$\lambda_{xy} = \frac{{\left| {P_{xy} (f)} \right|^{2} }}{{P_{xx} (f)P_{yy} (f)}}$$

where ${P}_{xy}$ presents the cross power spectral density of $x \left(t\right)$ and $y (t) ;$ ${P}_{xx}$ is the PSD of $x (t)$, and ${P}_{yy}$ is the PSD of $y (t)$. All these PSD could be calculated by Welch’s averaged modified periodogram method of spectral estimation. Coherence is a function with an independent variable $f$(frequency) describing the correlation of two signals at each frequency point $f$*.* The value of coherence function changes in the interval of [0, 1], and the value closer to 1 represents a stronger correlation between the two signals.

In this work, some typical values of coherence function around its peak frequency were extracted, and the average of these values was computed to explore how the loss of long synaptic projections effected the coherence. Further, the average values of coherence function around its peak frequency were defined as coherence, and the variation of coherence at different parameters of long synaptic projection was depicted in the following section.

## Main results

As pointed out by previous neuroanatomical research, the loss of connections furnished by long cortico-cortical projection neurons might result in AD^35^. In addition, the research of Xuereb et al.^36^ on neuronal system diseases also demonstrated that the number of synapses was reduced in the lesioned brain areas of AD. Moreover, the decreased synaptic number could lead to the loss of long synaptic projections. Therefore, in this section, the hallmark neuropathological condition of loss of long synaptic projections in AD was mimicked by decreasing the parameters of long synaptic projections in the model. Further, the effect of decreased long synaptic projections on coherence was explored by means of power spectral analysis and coherence function. Specifically, for descending projection of the dorsal visual pathway, the long synaptic projection from area v2 to v1 (${k}_{21}$), and that from area v5 to v2 (${k}_{52}$) were discussed. As for ascending pathway, a case was conducted on the long synaptic projection from area v2 to v5 (${k}_{25}$). The specific physiological background supporting the viewpoint that long synaptic projections (${k}_{21}$,${k}_{52}$ and ${k}_{25}$) were effected in AD was replenished at the beginning of each part afterwards.

### Loss of the long synaptic projection from area v2 to v1

**Figure 3:**
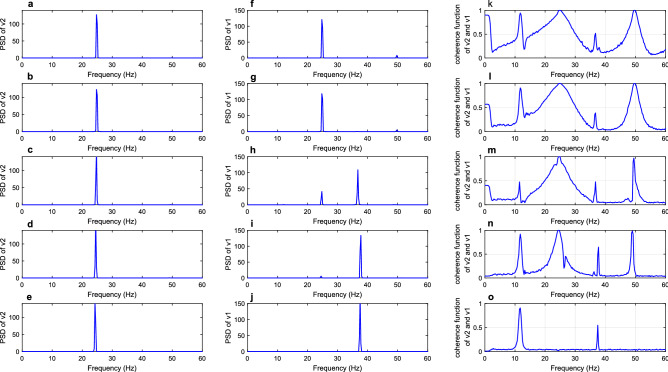
The first and second columns are PSD of areas v2 and v1, respectively. The coherence function between the two areas was delineated in the third column. From top to bottom, the parameters of long synaptic projection from v2 to v1 were in turn = 20, 15, 10, 5 and 0.

In the Ref.^37^, the research studied the laminar distribution of neurofibrillary tangles (NFTs) in visual cortex from AD patients, revealing that the total distribution of NFTs in layers III and V of area v2 is much denser than that in layers III and V of area v1. Neurons with NFTs play a major part in the loss of synapses^38^. Thus, it is reasonable to emulate the decreased long synaptic projection from area v2 to v1 in AD. In all simulations of this part, the parameters of long synaptic projections ${k}_{ij}$ were set to be 10 with the exception of ${k}_{21}$ (synaptic projection from area v2 to v1). For different values of ${k}_{21}$(${k}_{21}$= 20, 15, 10, 5, 0), Fig. 3 showed the PSD of areas v2 and v1, meanwhile, the coherence function between these two areas were also depicted here. Therein, the first and second columns depicted the PSD of areas v2 and v1, and the third column was the coherence function between areas v2 and v1. Firstly, area v1 received strong input from area v2 when the long synaptic projection from area v2 to v1 was set to be relatively greater values, such as ${k}_{21}$ = 20 and 15. As a consequence of this setting, the PSD of area v1 displayed one well-defined peak in the beta band with a maximum peak frequency of 24.75 Hz, which was consistent with the peak frequency of PSD of area v2, suggesting a strong relationship between areas v2 and v1. Meanwhile, the peak of coherence function reached 1 at 24.75 Hz, revealing the fact that the coherence between areas v2 and v1 in the beta range was very high. When the projection ${k}_{21}$ from v2 to v1 was reduced to 10, the power spectral peak of area v2 remained almost unchanged in the beta band, but the PSD of area v1 demonstrated another small peak located in the gamma band which was not originally present in the area v1. In the corresponding third column, the area enclosed by coherence function became narrow, suggesting a decline in the coherence between two areas in the beta band. Further, the peak in PSD of area v1 located in the beta band became small when ${k}_{21}$ dropped to 5, but the peak in PSD of area v1 located in the gamma band became more significant. Besides, the area enclosed by coherence function continued to decrease, indicating that the coherence between areas v2 and v1 also decreased. Finally, only one peak located in the gamma band could be seen in the PSD of area v1, and the peak originally located in the beta band disappeared as ${k}_{21}$ decreased to 0. Since the intrinsic rhythm of area v1 located in the gamma band, the rhythm of area v1 gradually approached its intrinsic rhythm, losing a similar rhythm to that of the area v2. Besides that, the peak of coherence function in the beta band decreased to a small value less than 0.1, which illustrated that the coherence between the two areas was very low.

**Figure 4:**
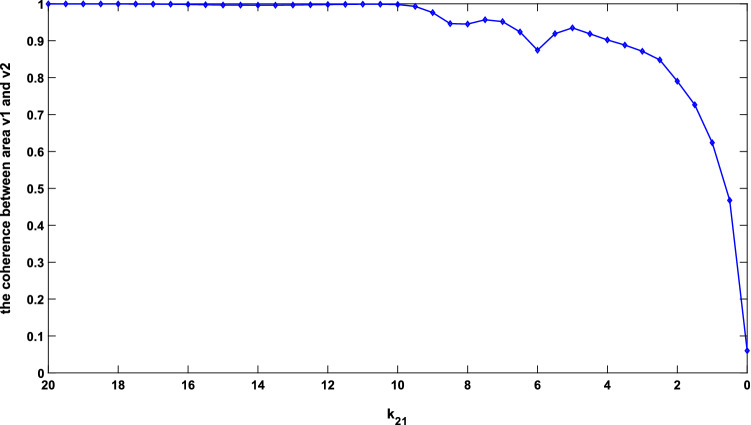
Coherence between areas v2 and v1 with ${k}_{21}$ changing from 20 to 0.

Since the coherence function reflected the correlation of two signals at each frequency point, the reduction in relation between the PSD of areas v2 and v1 in the beta band was consistent with the reduction of coherence function in the beta band. The above results reflect that the coherence between the two areas had a significant decrease with the decrease of long synaptic projection ${k}_{21}$. To verify this conclusion more comprehensively, the mean values of coherence function in the range of 24.5–25.25 Hz for different values of ${k}_{21}$ (${k}_{21}$ changed from 20 to 0) were computed. These mean values of coherence function were recorded as coherence which was depicted in Fig. 4 for different projection parameters. As shown in Fig. 4, the coherence remained nearly constant until ${k}_{21}$ dropped to about 10. As ${k}_{21}$ continued to decrease to zero, however, the coherence presented a downward trend and gradually minimized to a small value of less than 0.1. This downward trend of coherence within the beta band accorded with the experimental result of electrophysiology that coherence in the beta band tended to decrease in AD patients^12^, since the decrease of ${k}_{21}$ was used to simulate the loss of long synaptic projections which was a typical pathological hallmark in AD patients^35,38^.

### Loss of the long synaptic projection from area v5 to v2

Neuropathological research demonstrated much more NFTs in Brodmann area 19 than in Brodmann areas 17 and 18 for MCI and AD patients^39^ (In fact, area v1 is located in Brodmann area 17 while areas v2 and v5 in Brodmann areas 18 and 19, respectively). Further, NFTs as a histological hallmark of AD are closely related to long corticortical projection^37^. Thus, the long synaptic projections from area v5 are crucial when it comes to the effect of AD on the dorsal visual pathway.

**Figure 5:**
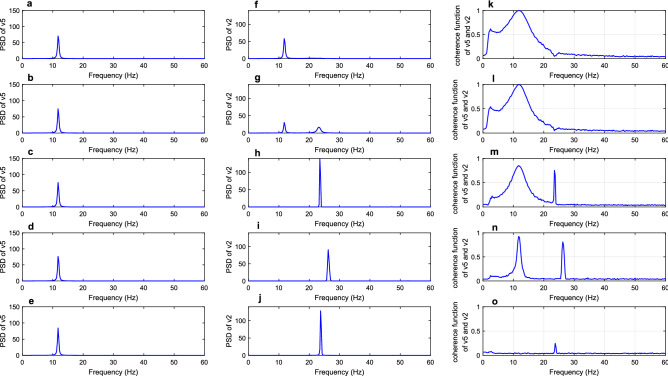
The first and second columns are PSD of areas v5 and v2, respectively. The coherence function between the two areas was delineated in the third column. From top to bottom, the parameters of long synaptic projection from v5 to v2 were in turn = 25, 20, 15, 5 and 0.

This part continued to discuss the descending projection of dorsal visual pathway and focused on the long synaptic projection ${k}_{52}$ from area v5 to v2. Firstly, the PSD of areas v5 and v2 and coherence function between areas v5 and v2 for different values of ${k}_{52}$ (${k}_{52}$= 25, 20, 15, 5 and 0) were delineated in Fig. 5. As ${k}_{52}$ was set to be 25 in the first line of Fig. 5, the PSD of area v2 displayed an evident peak in the alpha band with a maximum peak frequency of 11.75 Hz, which was consistent with the peak frequency of area v5. In addition, the peak of coherence function at 11.75 Hz reached a value of 1. These behaviors indicated high coherence between areas v5 and v2. When the projection ${k}_{52}$ from area v5 to v2 was reduced to 20, the PSD of area v2 exhibited two obvious peaks located in alpha and beta bands separately. It is worth noting that the intrinsic rhythm of area v2 was originally located in the beta band. Meanwhile, the area enclosed by coherence function around 11.75 Hz decreased, reflecting that the diminished long synaptic projection ${k}_{52}$ from area v5 to v2 led to decreased coherence in the alpha band. Further, the alpha rhythm of area v2 disappeared and area v2 showed just a single rhythm located in the beta band close to the intrinsic rhythm of area v2 as the value of ${k}_{52}$ fell to 15. Besides, the peak of coherence function in the alpha band decreased to approximately 0.8, indicating a reduction in coherence between areas v5 and v2. With ${k}_{52}$ continuing to decline to 5, the area enclosed by coherence function narrowed more. Finally, the PSD of area v2 had only one peak located in the beta band and the obvious peak of the PSD of area v5 was still located in the alpha band as ${k}_{52}$ decreased to 0. Meanwhile, the coherence function no longer displayed a peak in the alpha band, reflecting that the coherence between two areas in the alpha band was very low. The above results demonstrate that the PSD of areas v2 and v5 lost their direct relation gradually, and the coherence between the two areas had a significant decrease with the decrease of long synaptic projection ${k}_{52}$.

**Figure 6:**
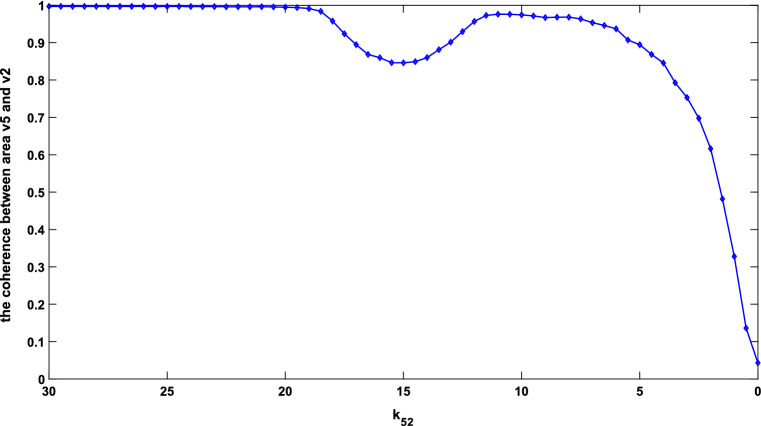
Coherence between areas v5 and v2 with ${k}_{52}$ changing from 30 to 0.

To explain this conclusion in more detail, the mean values of the coherence function in the range of 11.5–12 Hz for ${k}_{52}$ changing from 25 to 0 were computed and noted as coherence. Similarly, the dependence of coherence within the alpha band on ${k}_{52}$ is illustrated in Fig. 6. From this Fig. 6, the coherence kept properly flat until ${k}_{52}$ reached the value of about 20 and then showed a small fluctuation as ${k}_{52}$ varied from 20 to about 10. Finally, the coherence dropped to about zero. Thus, it can be concluded that the coherence between areas v5 and v2 showed a downward trend with long synaptic projection from area v5 to v2 becoming weaker. Since the neuropathological phenomenon of loss of long synaptic projections in AD was simulated by the decreasing strength of long synaptic projection ${k}_{52}$ in the present model^35,37,39^, the above result of a significant decrease in the alpha-band coherence was consistent with the experimental phenomena that alpha coherence was significantly decreased in AD patients^9,40,41^.

### Loss of the long synaptic projection from area v2 to v5

**Figure 7:**
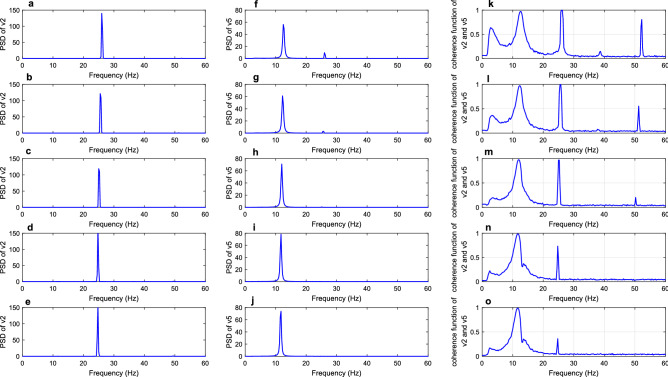
The first and second columns are PSD of areas v2 and v5, respectively. The coherence function between the two areas was delineated in the third column. From top to bottom, the parameters of long synaptic projection from v2 to v5 were in turn = 50, 35, 20, 5 and 0.

**Figure 8:**
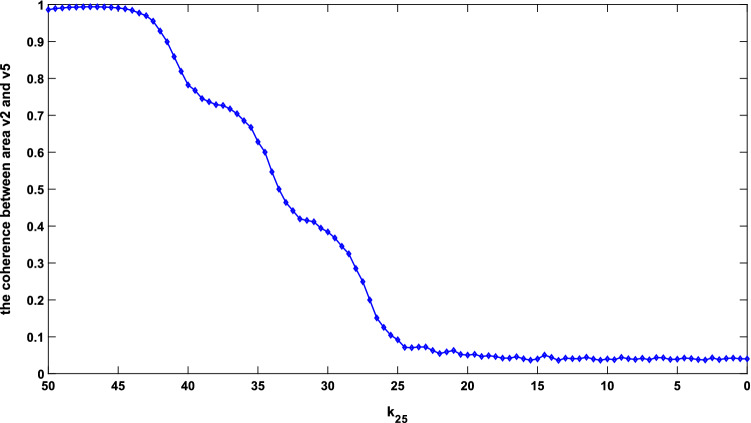
Coherence between areas v2 and v5 with ${k}_{25}$ changing from 50 to 0.

As for the ascending projection of dorsal visual pathway, the conclusion in Hof and Morrison^8^ presented that the long synaptic projection from area v2 to v5 might have significant lesions in AD. Thus, in this part, the effect of diminished long synaptic projection ${k}_{25}$ on the coherence of the model was discussed. The PSD of areas v2 and v5 and the corresponding coherence function for the specified value of ${k}_{25}$ (${k}_{25}$ = 50, 35, 20, 5, 0) were depicted in Fig. 7. From the first and second columns of this Fig. 7, it could be seen that the decrease in the long synaptic projection ${k}_{25}$ from area v2–v5 resulted in the loss of beta rhythm in area v5. However, the single rhythm of area v2 was always located in the beta band, indicating no direct relation between the PSD of areas v2 and v5. As for the coherence function between areas v2 and v5, the area enclosed by coherence function around 26 Hz within the beta band became narrow as ${k}_{25}$ decreased from 50 to 20. Then, the peak of coherence function in the beta band decreased to approximately 0.7 as the value of ${k}_{25}$ was 5. Finally, the peak of coherence function dropped to about 0.35 in the beta band and ${k}_{25}$ decreased to 0. This change suggested that the coherence between areas v2 and v5 was reduced by decreasing long synaptic projection ${k}_{25}$. To explain the change of coherence in more detail, the mean values of the coherence function in the range of 25.75–26.25 Hz for different values of ${k}_{25}$ were calculated. These mean values were recorded as the coherence, and the coherence at different projection parameters is depicted in Fig. 8. As shown in Fig. 8, the curve of coherence presented an S-shape, first remaining stable and then decreasing rapidly as ${k}_{25}$ varied from about 45–25. Finally, the coherence dropped and remained at a small value. This part mimicked the neuropathological phenomenon of lesion of the long synaptic projection from area v2 to v5 in AD by decreasing the value of ${k}_{25}$^8^, and the conclusion of decreased coherence in the beta band agreed with the experimental result of EEG in^12^.

## Conclusion and discussion

In light of the hierarchical organization in cerebral cortex and the information flows of the dorsal visual pathway, this study firstly constructed a biologically reasonable computational model on the dorsal visual pathway. This model included three visual cortical areas (areas v1, v2 and v5) which were interconnected by two types of long synaptic projections of ascending projection and descending projection. By reducing the parameters of the long synaptic projections to mimic the pathological condition of synaptic loss in AD, the effect of reduced long synaptic projections on coherence between different visual cortex areas was discussed using power spectral analysis and the coherence function. To be specific, through decreasing the long synaptic projections ${k}_{21}$ from area v2–v1, ${k}_{52}$ from area v5–v2 and ${k}_{25}$ from area v2–v5, we concluded that a significant reduction in the coherence occurred between areas v2 and v1, area v5 and v2, and area v2 and v5. These results obtained from the model of the dorsal visual pathway are consistent with the electrophysiological experimental result of EEG in AD patients^9,12,40,41^, which could be considered as a supplement to electrophysiological experiments and clinical diagnosis.

In this work, all simulations focused on the effect of reduced long synaptic projections on the coherence between cortical areas. Notably, coherence is a widely used measure of phase synchrony between pairs of signals, other metrics that can measure phase synchrony are phase-locking value (PLV) and pairwise phase consistency (PPC). For the PLV, it can be calculated by normalising the amplitude of the coherence^42^. For the PPC, it avoids the bias caused by the vector addition operation in PLV and is therefore less affected by the sample size^43^. In order to verify the robustness of the simulation results of this study under different phase synchrony measures, the PLV and the PPC were also used to measure the phase synchrony between areas v2 and v1, area v5 and v2, and area v2 and v5. The simulations showed a significant decrease in the PLV and the PPC between any two of these areas as long synaptic projections ${k}_{51}$, ${k}_{12}$ and ${k}_{15}$ reduced, which are consistent with the results using coherence method.

As described in the Introduction of this work, several experimental studies have proposed that AD may affect the dorsal visual pathway^4–8^. Further, some studies supported the idea that the dorsal visual pathway is more susceptible to neuropathological changes associated with AD than the ventral visual pathway. For example, the results of Bokde’s study suggested that the dorsal pathway was affected prior to neuronal function along the ventral pathway^44^. Gilmore’s study indicated that the dorsal visual pathway was more affected in AD and there was greater dysfunction along the dorsal visual pathway^45^. However, other studies that seem to support the idea that the ventral visual pathway is more affected in AD^46,47^. The ventral visual pathway includes areas vl, v2, v4, and inferior temporal areas TEO and TE, which is essential for the visual identification of objects^3^. Thus, constructing the model of ventral visual pathway to mimic the EEG dynamics observed in patients of AD will be explored in our future work.

Finally, it also should be noted that our model only focused on coherence between different cortical areas and did not consider the coherence at the level of single population. Since the neuron mass models derived from the Jansen’s model have the disadvantage of not being able to describe synchrony within a neuronal population. A model to better understand the changes in synchrony within a neuronal population is the next generation neural mass model proposed by Coombes and Byrne^48^. This model combines the theta-neuron model with the synaptic equations of the Jansen’s model, and the firing rate of the model is a function of the Kuramoto order parameter for measuring synchronicity. In this way the next generation neural mass model could simulate the phenomena of event-related desynchronisation and the event-related synchronisation within a single population model. This is a more realistic model with derived population firing rate related to synchrony rather than a phenomenological assumption. Therefore, the dynamical mechanisms of neural mass models to simulate decreased EEG coherence in AD will be explored in future researches, guided by the work of Coombes and Byrne.

In conclusion, the results of this study are significant for the comprehension of the decreased coherence in the EEG of AD. Moreover, the long synaptic projections from area v5 to v1, v1 to v2 and v1 to v5 were affected in AD as well. By reducing the parameters of long synaptic projections ${k}_{51}$, ${k}_{12}$ and ${k}_{15}$, a similar conclusion that the coherence between areas v5 and v1, v1 and v2 decreased could be drawn. For the lake of space, however, only the three typical synaptic projections were selected in this work to discuss their effects on coherence. We hope the results of this study could provide theoretical guidance for understanding the dynamic mechanisms of AD.

## Data Availability

The datasets used and/or analysed during the current study available from the corresponding author on reasonable request.
